# Evaluation of hemodialysis vascular access. Perspective from Mexico

**DOI:** 10.3389/fneph.2023.1084188

**Published:** 2023-07-11

**Authors:** Juan C. García-Yañez, Carlos A. Serrano-Gavuzzo, Mauricio Arvizu-Hernandez, Bernardo Moguel-González, Erick Bravo

**Affiliations:** ^1^ Department of Nephrology and Urology, High Specialty Regional Hospital of Bajio, León, Guanajuato, Mexico; ^2^ Department of Nephrology, Aranda de la Parra Hospital, Leon, Guanajuato, Mexico; ^3^ Department of Medicine and Nutrition, University of Guanajuato, Leon, Guanajuato, Mexico; ^4^ Department of Vascular Surgery, XXI Century National Medical Center, Mexican Social Security Institute, Mexico City, Mexico; ^5^ Department of Nephrology and Mineral Metabolism, Salvador Zubiran National Institute of Medical Sciences and Nutrition, Mexico City, Mexico; ^6^ Instituto Tecnológico y de Estudios Superiores de Monterrey, School of Medicine and Health Sciences, Mexico City, Mexico; ^7^ Department of Nephrology, Ignacio Chavez National Institute of Cardiology, Mexico City, Mexico; ^8^ Department of Vascular Surgery, Guadalupe Hospital, Celaya, Guanajuato, Mexico

**Keywords:** interventional nephrology, angiology and vascular surgery, vascular access, arterio venous fistula, Mexico, chronic kidney disease, hemodialysis, catheter

## Abstract

Chronic kidney disease (CKD) has become a global health problem. In 2019, it was related to 2.53% of general global mortality (2.35-2.66%); in the same year, in Latin America, mortality related to CKD reached 5.25% (4.92-5.49%), with an annual increase of 3.37%, proving increased mortality of 102% between 1990 and 2017. A nephrology specialty in Mexico recently fulfilled its first 50 years. Despite being relatively young, nephrologists are interested in “new” sub-specialties of nephrology and learning novel techniques and problem-solving skills. Our group is the first in our country to focus solely and exclusively on comprehensive VA care and we want to position ourselves as the first Mexican interdisciplinary group focused on vascular access (GIMEXAV).

## Introduction

Chronic kidney disease (CKD) has become a global health problem. For example, in 2019, it was related to 2.53% of general global mortality (2.35-2.66%); in the same year, in Latin America, mortality associated with CKD reached 5.25% (4.92-5.49%), with an annual increase of 3.37%, proving increased mortality of 102% between 1990 and 2017 (1). In Mexico, 9% of the population suffers from kidney disease, with considerable differences between the geographical regions. From 1990 to 2019, CKD-diabetes burden rapidly increased with the age-standardized years of life lost and disability-adjusted life-years (DALY) rates rising from 1,735.7 (1990) to 3,962.8 (2019), showing an increase of 128%. As a result, CKD-related mortality in Mexico reached 9.82% (9.29-10.22%) in 2019, with an annual rise of 4.85% (2).

To date, there is no national registry of CKD. Furthermore, due to the differences and disparities in health care in the country, where only about 58% of the population has access to social security (3), only this percentage of patients can freely access CKD treatments, including renal replacement therapy (RRT); about 52 million will not have access to RRT or will have to pay for it out of pocket even though for most of them this is unaffordable. In many cases, this difference in access to therapeutic options has an impact, increasing the mortality of patients with end stage kidney disease (ESKD), such as those reported three years after the start of RRT with a mortality of 38.2% in patients who had access to secure social security vs. 56.7% in patients without access to social security (4). According to the report by the Latin American Registry of Dialysis and Transplantation presented in 2019 and sponsored by the Latin American Society of Nephrology and Hypertension (SLANH), the prevalence of patients with RRT in 2013 reached 660 patients per million, an increase of more than 550% in 13 years. It is estimated that there are more than 400,000 patients with ESKD who receive some RRT; the most frequent is HD, which covers more than 50% of patients (>272,000 patients), followed by kidney transplantation, and, finally, peritoneal dialysis (5).

VA in patients with ESKD is a central management element and impacts patient comorbidities and mortality. Therefore, their planning, creation/placement, use, and surveillance are essential for daily management in this population and can be a frequent source of complications, ranging from episodes of dysfunction and infectious processes to death.

Related to the above, in 2019, the Kidney Disease Outcomes Quality Initiative (KDOQI) updated its vascular access guidelines, resuming an approach focused on the individual needs of the patient and comprehensive care for their Chronic Kidney Disease, this is known as PLAN (Patient Life-Plan: PL), followed by their corresponding access needs (AN) (6).

Considering data from the state of Aguascalientes and Jalisco with the total population of the country (126,014,024 as of 2020), the incidence of ESKD could represent around 60,864-71,820 patients, and the prevalence of RRT (Jalisco 1,050 pmp, Aguascalientes 1,352 pmp) would mean approximately 132,314-170,370 patients on RRT around the country (7); considering that between 54-65% are on hemodialysis (HD), this would represent a total of 71,449 to 110,740 patients on HD. However, there is no confirmation of these data by official sources.

There are few published data or records about the frequency of use of the different VA in patients treated with HD; in a report presented at a scientific meeting in 2014, the epidemiology of 13,373 patients in 23 HD units was that more than 74% of patients started RRT with a catheter, 65% with a temporary non-tunneled catheter, 10.5% with a tunneled catheter, 20.5% with an arteriovenous fistula (AVF), and 1.5% with a vascular graft (8).

Another report from the National Academy of Medicine described the frequency of VAs in 955 chronic HD patients, reporting that 52% of the patients had a catheter and 48% had an AVF (9).

However, in a study of 818 patients in 83 HD units, it was found that only 8% of patients had an AVF (10).

It is essential to recognize the high frequency in the country of the use of non-tunneled catheters as the first VA in more than 80% of patients, and many of them (> 90%) can remain with this VA after the first month of treatment or even longer extended periods (11).

In addition, VA care is diverse; in a report from a Mexican Social Security Institute HD unit with 36 chronic patients of which only 33% of patients had an AVF, both groups of patients—AVF and catheters—had low levels of knowledge of VA self-care (12). There have been some reports regarding the survival of patients with VAs in the country; the results of 692 patients have been analyzed, where 143 AVFs (20%) were created, and a primary failure rate of 16.7% was observed; when comparing AVFs against right jugular tunneled-catheters, a better survival was observed in AVFs at one year (AVF 94% vs. 81% TC) and at two years (AVF 90% vs. 77% TC), with similar survival rates between younger and older patients over 55-years-old (13).

Given this background, it is necessary to promote a national registry of patients with ESKD that considers VA management and encourages policies for better use of VA for HD at all levels of care.

## Epidemiology of VA in Mexico

**Figure 1:**
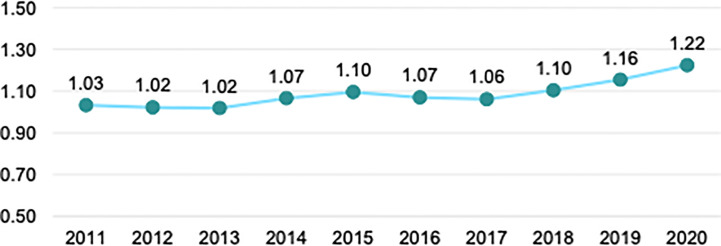
Rate of registered deaths from CKD per 10,000 population.

In 2020, patient deaths from CKD reached 15,455 registered deaths. ESKD accounted for 74% of all deaths from kidney disease, according to the National Institute of Statistics, Geography, and Informatics (INEGI), with the highest incidence in people over 45 years, particularly men (14). The following graph shows that from 2011 to 2020, CKD deaths have increased steadily (**Figure 1**).

It is estimated that 70% of the population in Mexico is overweight; of these, a third is obese, which is considered a primary risk factor for diabetes mellitus. It is estimated that around 6.2 million Mexicans with diabetes mellitus have CKD in different stages. The prevalence of patients with CKD is 12.2%, and 51.4 deaths per 100,000 population with an incidence of 458 and an increase in incidence from 16% to 38%, and an increase in prevalence from 2006 to 2016 of 30% to 45%, respectively. The economic impact of CKD was estimated for 2014 at $8,966 USD per patient, according to the National Institute of Public Health (NIPH) (15).

In Latin America, only Brazil, Argentina, Chile, Cuba, Uruguay, Venezuela, and Colombia provide universal access to RRT. However, the number of health providers (nephrologists, nurses, HD technicians, transplant surgeons, angiologists, and vascular surgeons) is insufficient for the health demands of this disease in Mexico. In addition, this annual expense represents a health cost for the patient, family members, and institutions and is a challenge for kidney transplantations and HD units.

The Official Mexican Standards (NOM) are technical regulations of mandatory observance issued by the competent agencies, whose purpose is to establish the characteristics that the processes or services must meet when they may constitute a risk to the safety of people or harm human health, as well as those related to terminology and those that refer to their compliance and application.

The official Mexican standard for the practice of HD, NOM-003-SSA03-2010, still does not contemplate HD VA (16). Our group agrees with the sequence that VA should be the first choice in Mexico in the AVF with native vessels, followed by AVFs with grafts, and finally, long-term catheters.

Hinojosa et al., “Actions in favor of vascular access for hemodialysis in Mexico" (17) presents an updated reference on the current state of VA, “ presents an updated reference on the current state of VA,” where the authors maintain a percentage of AVF of 48%, 73% of the prevalent population on HD have social security. However, these data were only obtained by survey and would require broader statistical analysis.

The creation of VA by angiologists and vascular surgeons in Mexico is influenced by North American and European guidelines rather than by a practical guide based on the needs of the Mexican population. Our group agrees with the concepts and positions of the KDOQI guidelines in carrying out the Life-Plan for ESKD.

## Conclusion

We need to include more training and practice on VA for HD in specialist training programs. Institutions with a vascular access team (VAT) are more likely to use tools, algorithms, technology, and guidelines to ensure success and decrease procedure complications. Teamwork is essential for the benefit of the patient.

We are trying to start a working group with the addition of nephrologists and vascular surgeons and establish the statements and local guidelines for VA in ESKD patients.

## Data availability statement

The original contributions presented in the study are included in the article/supplementary materials. Further inquiries can be directed to the corresponding author.

## Ethics statement

Ethical review and approval was not required for the study of human participants in accordance with the local legislation and institutional requirements. Written informed consent from the participants was not required to participate in this study in accordance with the national legislation and the institutional requirements.

## Author contributions

All authors listed have made a substantial, direct, and intellectual contribution to the work and approved it for publication.
